# The Importance of Tight f Basis Functions for Heavy p-Block Oxides and Halides: A Parallel With Tight d functions in the Second Row

**DOI:** 10.1021/acs.jpca.3c00544

**Published:** 2023-02-28

**Authors:** Nisha Mehta, Jan M. L. Martin

**Affiliations:** Department of Molecular Chemistry and Materials Science, Weizmann Institute of Science, 76100 Reḥovot, Israel

## Abstract

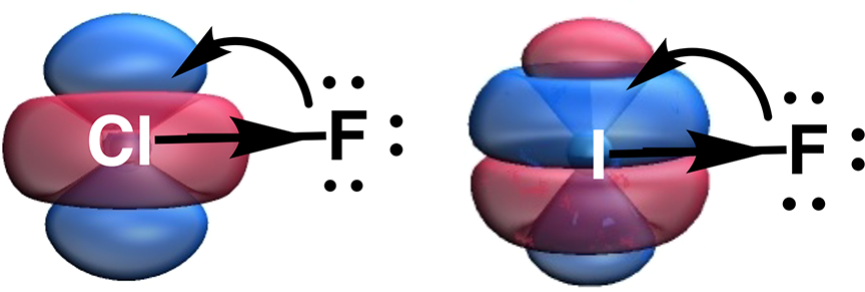

It is well-known that both wave function ab initio and DFT calculations on second-row compounds exhibit anomalously slow basis set convergence unless the basis sets are augmented with additional “tight” (high-exponent) d functions, as in the cc-pV(n+d)Z and aug-cc-pV(n+d)Z basis sets. This has been rationalized as being necessary for a better description of the low-lying 3d orbital, which as the oxidation state increases sinks low enough to act as a back-donation acceptor from chalcogen and halogen lone pairs. This prompts the question whether a similar phenomenon exists for the isovalent compounds of the heavy p-block. We show that for the fourth and fifth row, this is the case, but this time for tight f functions enhancing the description of the low-lying 4f and 5f Rydberg orbitals, respectively. In the third-row heavy p block, the 4f orbitals are too far up, while the 4d orbitals are adequately covered by the basis functions already present to describe the 3d subvalence orbitals.

## 1 Introduction

When, in the early 1990s, the G1 and G2 computational thermochemistry approaches^1^ were extended to second-row elements,^2,3^ SO_2_ was found to be a significant outlier. The G1 team found that adding a third set of d functions to the basis set increased the atomization energy of SO_2_ by 8 kcal/mol; they ascribed this to hypervalence. Indeed in G4 theory,^46^ an additional layer of d functions is placed on Al-Cl.

Bauschlicher and Partridge^4^ studied basis set convergence for SO_2_ in detail for both CCSD(T) and the B3LYP density functional approach, and at both levels found hypersensitivity to high-exponent d functions.

Martin,^5^ in 1998, showed that this is the case also for other properties such as vibrational frequencies, as well as that nearly the entire contribution in CCSD(T) can be ascribed to the HF-SCF component of the energy. Moreover, this persisted when the inner-shell orbitals were replaced by an effective core potential, refuting claims that core polarization might be involved.

Later benchmark studies on second-row molecules found more severe examples in SO_3_ (40 kcal/mol)^6^ and in perchloric acid and perchloric anhydride (50 and 100 kcal/mol, respectively).^7^ It was also found (see ref (7) for a discussion) that the strength of the effect was roughly proportionate to the formal oxidation state of the central second-row atom: NBO (natural bond orbital) analysis^8^ revealed^7^ that, as the central atom becomes more positively charged, the 3d orbitals sink lower and become ever more available for back-donation from chalcogen and halogen lone pairs.

The textbook concept of hypervalence, e.g., d^2^sp^3^ hybridization in SF_6_ which violates the octet rule, has been comprehensively refuted by Reed and Schleyer^9^ and by Cioslowski and Mixon^10^ (see Norman and Pringle^11^ for a recent review, as well as section 7.4 of Schwerdtfeger, Frenking, and co-workers^12^). One of us has, however, referred^13^ to 3d orbitals in such molecules as “honorary valence orbitals of the second kind”. (The “first kind” in that paper refers to subvalence orbitals that are energetically so close to the valence shell that freezing them in correlated calculations may cause catastrophic errors.^14,15^)

In response to the findings described above, the correlation consistent basis sets for second-row elements have been revised^16^ to include additional “tight” (i.e., high-exponent) d functions, giving rise to cc-pV(n+d)Z and aug-cc-pV(n+d)Z basis sets.

The authors have both been asked whether this situation is unique to second-row elements, and if yes, why. We shall show below that, in fact, a similar phenomenon occurs for 4f orbitals in the fourth-row heavy p-block elements—the last p-row of the periodic table before the lanthanides—as well as for 5f orbitals in the fifth-row heavy p-block—the last p-row before the actinides. To the best of our knowledge, the first work to refer to the need for tight f functions was the 2005 Weigend–Ahlrichs paper defining the “def2” basis sets.^17^ Studies by Dixon et al.^18^ on iodine fluorides and their ions, and by Peterson^19^ on IOO^–^, both reference this same necessity, while Hill and Peterson,^20^ in the paper introducing the cc-VnZ-PP-F12 basis sets for the heavy p-block elements, add 1d1f tight functions for the same reason as well as for describing outer-core correlation. However, while the need for tight f functions on heavy p-block elements has been pointed out prior to this work, it is safe to say it is not broadly known in the theoretical chemistry community and has not been investigated systematically.

## 2 Methods

In the present work, we investigate the effect of tight d and f functions on the total atomization energies of heavy p-block oxides and halides at the HF, DFT, and CCSD(T)^21,22^ level of theories. All geometries are optimized at the PW6B95^23^-D3(BJ)^24^/def2-QZVPP^17^ level of theory, and XYZ coordinates are reported in the Supporting Information. All calculations are carried out using the Gaussian 16^25^ and MOLPRO^26^ Program suites, running on the Faculty of Chemistry HPC facility “ChemFarm” at the Weizmann Institute. We considered the correlation consistent basis sets of Dunning and co-workers.^27,28^ In this paper, we considered aug-cc-pVnZ^29^ and aug-cc-pV(n+d)Z^16^ basis sets for second-row elements and aug-cc-pVnZ-PP^30,31^ for heavy p-block elements, where PP stands for the Stuttgart–Cologne energy-consistent relativistic pseudopotentials.^32^ We also considered weighted core–valence basis sets: aug-cc-pwCVnZ^29,33^ for second-row and aug-cc-pwCVnZ-PP^30,31,34^ for heavy p-block elements. For brevity, aug-cc-pVnZ(-PP), aug-cc-pV(n+d)Z, and aug-cc-pwCVnZ(-PP) basis sets are denoted as aVnZ(-PP), aV(n+d)Z, and awCVnZ(-PP), respectively. The high-exponent (d,f) functions that are added to the aVnZ(-PP) basis set are taken from the core–valence awCVnZ(-PP) basis sets for the respective elements. All calculations are carried out within the frozen core approximation with Gaussian’s int(grid = ultrafine), which corresponds to the pruned direct product of a 99-point Euler-Maclaurin radial and a 590-point Lebedev angular grid or its MOLPRO counterpart. The default tight SCF convergence criterion was used, which corresponds to the norm of the density matrix update being <10^–8^ and the largest individual element change being <10^–6^ in absolute value. We also carried out natural bond orbital (NBO) population analysis^8^ at the Hartree–Fock and DFT levels using the NBO7 program^35^ interfaced to both Gaussian 16^25^ and MOLPRO.^26^ As Gaussian has no CCSD(T) analytical derivatives and hence cannot write out first-order reduced density matrices at the CCSD(T) level, we assessed the importance of (T) for the NBO populations using MOLPRO, both using its built-in NBO implementation and using NBO7 via the interface. The differences between CCSD and CCSD(T) NBO populations were found to be negligible. The BFGS algorithm^36^ as implemented in MOLPRO 2022 is used for the exponent optimizations, with the gradient threshold set to 10^–5^.

## 3 Results and Discussion

### 3.1 Effect of Tight d and f Functions on the Hartree–Fock Component

Table 1 presents the difference between the atomization energies calculated with the aVTZ(-PP) basis set and those obtained by adding a progressively large set of (d,f) functions to the basis set at the Hartree–Fock level. As it was previously found (e.g., Section 2.2 in ref (37)) for the second-row elements that core–valence basis functions are already in the right exponent range, we assumed that the tight d and f exponents from the cc-pwCVnZ basis sets were a reasonable starting point.

**Table 1:** Difference between the Hartree–Fock Component of the Total Atomization Energy Obtained with the aVTZ(-PP) and aVTZ(-PP)+(d,f) Basis Sets [aVTZ(-PP)-aVTZ(-PP)+(d,f) or awCVTZ-PP in kcal/mol]^a^

		Central atom			Central atom			Central atom			Central atom	
		Rydberg			Rydberg			Rydberg			Rydberg	
	ΔSCF	f	d	ΔSCF	f	d	ΔSCF	f	d	ΔSCF	f	d
	ClO_4_^–^			SO_3_			PF_5_					
aVTZ	REF	0.0097	0.2806	REF	0.0107	0.1885	REF	0.0163	0.1226			
aV(T+d)Z	21.20	0.0095	0.3220	14.94	0.0106	0.2176	9.53	0.0161	0.1382			
aVTZ+1d	20.29	0.0095	0.3206	14.50	0.0105	0.2159	9.26	0.0160	0.1373			
aVTZ+2d	23.57	0.0095	0.3275	16.67	0.0106	0.2207	10.65	0.0161	0.1401			
aVTZ+1f	0.72	0.0102	0.2805	0.71	0.0108	0.1884	1.12	0.0167	0.1226			
aVTZ+2d1f	24.34	0.0100	0.3275	17.44	0.0107	0.2207	11.81	0.0165	0.1401			
awCVTZ	24.49	0.0101	0.3265	17.52	0.0104	0.2202	11.89	0.0160	0.1395			
	BrO_4_^–^			SeO_3_			AsF_5_			KrF_6_		
aVTZ-PP	REF	0.0185	0.1367	REF	0.0163	0.0841	REF	0.0187	0.0521	REF	0.0153	0.1379
aVTZ-PP+1d	0.67	0.0186	0.1388	0.49	0.0163	0.0859	0.34	0.0188	0.0533	0.29	0.0153	0.1394
aVTZ-PP+2d	0.73	0.0187	0.1395	0.51	0.0164	0.0866	0.37	0.0188	0.0537	0.35	0.0153	0.1396
aVTZ-PP+1f	1.54	0.0202	0.1364	1.19	0.0171	0.0839	1.65	0.0199	0.0520	0.38	0.0159	0.1378
aVTZ-PP+2d1f	2.30	0.0203	0.1393	1.73	0.0172	0.0865	2.05	0.0200	0.0536	0.74	0.0159	0.1396
aVTZ-PP+2f	1.62	0.0204	0.1364	1.24	0.0172	0.0839	1.71	0.0201	0.0520	0.43	0.0159	0.1378
aVTZ-PP+2d2f	2.38	0.0205	0.1393	1.78	0.0173	0.0865	2.11	0.0201	0.0535	0.78	0.0159	0.1396
awCVTZ-PP	2.80	0.0203	0.1384	2.16	0.0171	0.0859	2.97	0.0197	0.0530	0.98	0.0161	0.1398
	IO_4_^–^			TeO_3_			SbF_5_			XeF_6_		
aVTZ-PP	REF	0.0416	0.0842	REF	0.0261	0.0529	REF	0.0222	0.0280	REF	0.0343	0.1022
aVTZ-PP+1d	0.08	0.0419	0.0867	0.08	0.0262	0.0545	0.10	0.0222	0.0288	0.03	0.0343	0.1046
aVTZ-PP+2d	0.16	0.0420	0.0879	0.09	0.0263	0.0552	0.12	0.0223	0.0290	0.13	0.0343	0.1059
aVTZ-PP+1f	7.98	0.0554	0.0829	4.47	0.0325	0.0524	5.31	0.0288	0.0276	3.95	0.0426	0.1015
aVTZ-PP+2d1f	8.15	0.0558	0.0866	4.58	0.0326	0.0547	5.42	0.0288	0.0284	4.07	0.0426	0.1052
aVTZ-PP+2f	11.36	0.0660	0.0824	5.88	0.0367	0.0523	6.71	0.0320	0.0275	6.63	0.0503	0.1009
aVTZ-PP+2d2f	11.52	0.0663	0.0861	5.98	0.0368	0.0546	6.83	0.0320	0.0285	6.75	0.0503	0.1047
awCVTZ-PP	12.72	0.0660	0.0852	7.28	0.0367	0.0542	9.55	0.0318	0.0280	8.09	0.0507	0.1049
	AtO_4_^–^			PoO_3_								
aVTZ-PP	REF	0.0439	0.0434	REF	0.0258	0.0261						
aVTZ-PP+1d	0.07	0.0442	0.0456	0.02	0.0259	0.0275						
aVTZ-PP+2d	0.20	0.0442	0.0462	0.08	0.0259	0.0279						
aVTZ-PP+1f	8.38	0.0608	0.0425	4.76	0.0336	0.0258						
aVTZ-PP+2d1f	8.55	0.0610	0.0453	4.84	0.0337	0.0275						
aVTZ-PP+2f	9.98	0.0673	0.0424	5.49	0.0366	0.0257						
aVTZ-PP+2d2f	10.15	0.0675	0.0452	5.57	0.0367	0.0274						
awCVTZ-PP	11.73	0.0675	0.0447	7.16	0.0367	0.0270						

a The NPA population of the d and f Rydberg orbitals at the Hartree–Fock level for the given basis set is also reported.

First, we analyze the XO_4_^–^ series (where X = Cl, Br, I, and At). The effect of adding tight d functions to the aVTZ basis set is quite significant for ClO_4_^–^, as well-documented:^7^ for instance, adding one d function to the aVTZ basis set increases the atomization energy of ClO_4_^–^ (i.e., ΔTAE_SCF_) by 20.29 kcal/mol. The corresponding values for adding progressively larger sets of tight (d,f) functions to the aVTZ basis set are 23.57 (+2d), 0.72 (+1f), and 24.34 (+2d1f) kcal/mol. Therefore, the large TAE increase seen for aug-cc-pwCVTZ (24.49 kcal/mol) is almost entirely due to the effect of d functions on the Cl atom. Table 1 also lists the overall population of the d and f Rydberg orbitals taken from the NBO population analysis of the HF determinant. (This can be obtained either by modifying the NBO7 source code to print more than two decimal places in the Natural Electron Configuration section of the output or by means of a simple shell and awk script that searches for the Rydberg NAO occupations of a given angular momentum and sums them up.) An NBO analysis of the wave function reveals that the natural population of chlorine d orbitals (*q*_3d_) increases as much as 0.05 when tight d functions are added. Specifically, *δq*_3d_ values, that is, the changes in the NPA populations relative to the aVTZ basis set, are 0.041, 0.040, and 0.047, respectively, for aV(T+d)Z, aVTZ+1d, and aVTZ+2d. As discussed in the Introduction, the chemical significance of the tight d functions to the aVTZ basis set is that they increase the ability of the chlorine 3d orbitals (in ClO_4_^–^) to act as back-bonding acceptors. The chlorine 4f orbitals are too far up in energy to participate significantly. These findings are consistent with those reported in ref (7) for Cl_2_O_7_ and HClO_4_.

The effect of high-exponent d functions is drastically reduced for third-row pseudohypervalent molecules (e.g., BrO_4_^–^). Adding two high-exponent d functions to the aVTZ-PP basis set affects the SCF component of the total atomization energy by a measly 0.73 kcal/mol, compared to 23.57 kcal/mol for ClO_4_^–^. Likewise, *q*_4d_ is affected by only 0.003. Furthermore, inclusion of tight f functions (i.e., aVTZ-PP+2f) has a negligible effect, and the HF-SCF component of the total atomization energy is affected by just 1.62 kcal/mol, which is considerably less than other sources of basis set incompleteness error for aVTZ-PP. Furthermore, the 4f_pop_ population is essentially unchanged. In the case of BrO_4_^–^, the bromine 3d orbitals are filled. Bromine 4d orbitals can act as back-bonding acceptors, but enough high-exponent d functions are already present in the underlying basis set (for the purpose of describing the 3d subvalence orbital); hence, the additional high-exponent d functions have a negligible contribution. The 4f orbitals are still too far up; hence, they do not benefit from the additional tight f functions.

Next, what happens when high-exponent (d,f) functions are added to the aVTZ-PP basis set for the IO_4_^–^ molecule? Analogous to perbromate, adding tight d functions for IO_4_^–^ affects the HF-SCF(TAE) value by less than 0.2 kcal/mol, making them chemically insignificant. The situation for high-exponent f functions is starkly different: TAE_SCF_ values are increased by 7.98 (aVTZ-PP+1f) and 8.15 (aVTZ-PP+2f) kcal/mol, with a concomitant *q*_4f_ increase of ≈0.02. While the iodine 4d orbitals are filled, 5d and 4f orbitals have nontrivial occupations. Similarly to BrO_4_^–^, there are enough decontracted functions from the valence shell of the basis set for the iodine 5d orbitals, but extra primitives are indeed necessary for 4f orbitals. Analogous to 3d orbitals in ClO_4_^–^, the addition of extra high-exponent f functions on iodine increases the ability of low-lying virtual 4f orbitals to act as back-bonding acceptors from chalcogen and halogen lone pairs.

The effect of high-exponent f functions is even more pronounced in perastatate, AtO_4_^–^. For instance, TAE[AtO_4_^–^] is increased by appreciable amounts of 8.38 (aVTZ-PP+1f) and 9.98 (aVTZ+2f) kcal/mol. The extra f functions increase *q*_5f_ on the At atom by ≈0.02. As expected, only trivial increases are seen for aVTZ+1d (0.07 kcal/mol) and aVTZ+2d (0.20 kcal/mol) basis sets. While the valence 5d orbitals are filled, 6d and 5f orbitals have significant occupations. Again, while there are enough decontracted functions for 6d orbitals from the valence shell of the basis set, extra high-exponent f functions are needed for the description of 5f orbitals.

We can summarize the above findings by saying that, for p-block elements in higher oxidation states, the second row requires tight d functions, while the fourth and fifth rows require tight f functions, and the third row requires neither. The fact that the second-row heavy p-block is approaching the first-row transition elements, and the fourth and fifth row heavy p-blocks the lanthanide and actinide series, respectively, is not a coincidence but directly linked to the energetic proximity of the d and f Rydberg orbitals.

For systems having lower oxidation states on the central atom, proportionally smaller effects are observed. For ClO_3_^–^ and ClO_2_^–^, the HF-SCF component of the total atomization energy is affected by 12.909 and 5.113 kcal/mol, respectively, whereas for IO_3_^–^, AtO_3_^–^, IO_2_^–^, and AtO_2_^–^, the effect of the tight f functions ranges from 2.665 to 7.250 kcal/mol (Table 2).

**Table 2:** Effect of Tight d and f Functions on the HF-SCF Component of the TAE (kcal/mol) as a Function of the Oxidation State of the Central Halogen Atom

	aVnZ vs aV(n+d)Z, aVnZ-PP vs aVnZ-PP+1f					
	n = D		n = T		n = Q	
	d	f	d	f	d	f
ClO_4_^–^	40.408		21.205		12.091	
ClO_3_^–^	24.087		12.909		7.448	
ClO_2_^–^	9.420		5.113		3.013	
ClO^–^	2.140		1.156		0.698	
BrO_4_^–^	7.178	1.730	0.672	1.542	0.105	0.500
BrO_3_^–^	4.646	1.379	0.405	1.174	0.082	0.424
BrO_2_^–^	1.825	0.599	0.151	0.502	0.042	0.200
BrO^–^	0.460	0.185	0.033	0.148	0.014	0.067
IO_4_^–^		8.882		7.984		4.073
IO_3_^–^		6.763		6.136		3.064
IO_2_^–^		2.892		2.665		1.343
IO^–^		0.908		0.829		0.419
AtO_4_^–^		14.110		8.384		4.295
AtO_3_^–^		11.650		7.250		3.599
AtO_2_^–^		4.936		3.105		1.545
AtO^–^		1.566		0.972		0.482

What happens to the equilibrium distances and harmonic frequencies when extra (d,f) functions are added? Table 3 contains the computed *r*(X–O) bond distances and vibrational frequencies for XO_4_^–^ (where X = Cl, Br, I, and At) obtained by progressively adding (d,f) functions to the aVTZ basis set. There are only four unique vibrational frequencies: T_2_ (triply degenerate bend), E (degenerate bend), A_1_ (symmetric stretch), and T_2_ (triply degenerate asymmetric stretch). Adding just one d exponent shortens the *r*(Cl–O) distance by 0.013 Å, and consequently,^38,39^ the vibrational frequencies are blue-shifted by 3–4% (16.6, 23.3, 42.9, and 31.0 cm^–1^, respectively). aVTZ+2d further raises the vibrational frequencies by 2.5, 3.6, 6.9, and 5.2 cm^–1^. The addition of high-exponent f functions, on the other hand, has an insignificant impact on *r*(Cl–O) distance and vibrational frequencies, as expected.

**Table 3:** Effect of Tight (d,f) Functions on Equilibrium Bond Distances (Å) and Harmonic Frequencies (cm^–1^) at the HF Level

ClO_4_^–^							
	aVTZ	aVTZ+1d	aVTZ+2d	aVTZ+1f	aVTZ+2d1f	awCVTZ	aV(T+d)Z
ω_1_(E)	502.3	518.9	521.4	503.4	522.7	522.5	519.3
ω_2_(T_2_)	696.0	719.3	722.9	697.2	724.3	724.2	720.1
ω_3_(A_1_)	1034.6	1077.5	1084.4	1035.7	1085.4	1085.3	1080.4
ω_4_(T_2_)	1212.5	1243.5	1248.7	1212.6	1248.5	1247.9	1246.9
*r*(Cl–O)	1.428	1.415	1.412	1.427	1.412	1.412	1.414

BrO_4_^–^								
	aVTZ-PP	aVTZ-PP+1d	aVTZ-PP+2d	aVTZ-PP+1f	aVTZ-PP+2d1f	aVTZ-PP+2f	aVTZ-PP+2d2f	awCVTZ-PP
ω_1_(E)	383.1	383.7	383.7	385.1	385.8	385.1	385.9	386.1
ω_1_(T_2_)	486.5	487.4	487.5	488.2	489.2	488.2	489.2	489.5
ω_1_(A_1_)	920.0	921.6	921.7	922.9	924.6	923.1	924.8	925.6
ω_1_(T_2_)	1012.7	1013.5	1013.7	1014.9	1015.8	1015.1	1015.9	1016.3
*r*(Br–O)	1.578	1.577	1.577	1.575	1.575	1.575	1.574	1.574

IO_4_^–^								
	aVTZ-PP	aVTZ-PP+1d	aVTZ-PP+2d	aVTZ-PP+1f	aVTZ-PP+2d1f	aVTZ-PP+2f	aVTZ-PP+2d2f	awCVTZ-PP
ω_1_(E)	301.9	302.0	302.1	307.7	307.8	309.5	309.6	309.8
ω_2_(T_2_)	368.0	368.1	368.2	371.5	371.8	372.6	372.9	373.0
ω_3_(A_1_)	890.5	890.5	890.7	902.5	902.5	907.5	907.5	909.4
ω_4_(T_2_)	956.2	956.0	956.2	967.6	967.4	972.5	972.3	972.9
*r*(I–O)	1.747	1.747	1.747	1.737	1.736	1.733	1.732	1.731

AtO_4_^–^								
	aVTZ-PP	aVTZ-PP+1d	aVTZ-PP+2d	aVTZ-PP+1f	aVTZ-PP+2d1f	aVTZ-PP+2f	aVTZ-PP+2d2f	awCVTZ-PP
ω_1_(E)	251.0	251.0	251.1	256.8	256.9	257.7	257.7	257.9
ω_2_(T_2_)	281.6	281.6	281.7	284.3	284.4	284.6	284.7	285.1
ω_3_(A_1_)	760.5	760.5	760.7	774.9	774.9	777.4	777.4	780.8
ω_4_(T_2_)	800.9	800.7	801.0	813.6	813.4	815.9	815.7	817.8
*r*(At–O)	1.866	1.866	1.866	1.851	1.851	1.848	1.848	1.846

Proceeding to BrO_4_^–^, the addition of inner polarization d and/or f functions has a modest effect on *r*(Br–O) bond distances and vibrational frequencies (just 2–3 cm^–1^).

In contrast, for the iodine and astatine oxides, adding a high-exponent f function blue-shifts all frequencies, by 5.8, 3.5, 12, and 11.4 cm^–1^ for IO_4_^–^ and by 5.8, 2.7, 14.4, and 12.7 cm^–1^ for AtO_4_^–^. Adding a second hard f function still contributes 1.8, 1.1, 5, and 4.9 cm^–1^ for IO_4_^–^, which is somewhat significant for the stretching frequencies, and 0.9, 0.3, 2.5, and 2.3 cm^–1^ for AtO_4_^–^. The tight d exponent contributions for IO_4_^–^ and AtO_4_^–^ molecules are essentially nil.

As expected, and consistent with the lower oxidation states of the central atoms, for the chalcogen and pnictogen based molecules SO_3_, SeO_3_, TeO_3_, PoO_3_, PF_5_, AsF_5_, and SbF_5_, we obtain somewhat milder effects of the tight (d,f) functions. Consistent with the halogen series, the addition of two high-exponent d functions increases the atomization energy of SO_3_ by 16.67 kcal/mol; for PF_5_, the magnitude of the effect is 10.65 kcal/mol. The contribution of the hard d exponent dwindles from right to left in the third row of the Periodic Table: from 0.49 (SeO_3_) to 0.34 kcal/mol (AsF_5_). Note also that adding a second hard d function contributes negligibly (0.03 kcal/mol). For TeO_3_, PoO_3_, and SbF_5_, as advocated in the previous section, the high-exponent f functions’ contribution increases rapidly for the third and fourth-row pseudohypervalent molecules. The addition of one high-exponent f function increases the total atomization energy of TeO_3_ and SbF_5_ by around 5 kcal/mol; for two f functions, the magnitude of effect is further increased by 1–2 kcal/mol.

A remark is in order about the noble gas fluorides (e.g., KrF_6_ and XeF_6_). Consistent with BrO_4_^–^, little effect is seen (see Table 1) from addition of either tight d or f functions to the Kr aVTZ-PP basis set. For XeF_6_ in aVTZ-PP, on the other hand, the first added tight f function accounts for 3.95 kcal/mol at the HF level. (As expected, we find that additional tight d functions have no impact worth speaking of.) Adding the next f function increases TAE_SCF_ by 2.68 kcal/mol: clearly, this effect is not specific to halogens.

In order to verify that our conclusions are not due to an artifact of our choice of exponents (i.e., tight d and f functions taken from the core–valence basis sets for the respective elements), we also reoptimized them at the HF level in the respective perhalides ClO_4_^–^, BrO_4_^–^, IO_4_^–^, and AtO_4_^–^. The exponents we found are reported in Table 4. Our main point remains unchanged; however, for higher angular momentum basis sets, such as aVQZ-PP and aV5Z-PP, a single optimized f exponent recovers nearly as much energy as two fixed ones from the awCVQZ(-PP) and awCV5Z-PP basis sets, respectively, especially for AtO_4_^–^. For aVTZ-PP basis set, the exponents we found are actually, by and large, close to those obtained from the awCVTZ-PP basis set.

**Table 4:** Comparison of Optimized Tight f Exponents with Those Taken from the Core–Valence Basis Sets of Iodine and Astatine and Comparison of ΔTAE

			ΔTAE (kcal/mol)			ΔTAE (kcal/mol)	
		exponent	HF	CCSD(T)	exponent	HF	CCSD(T)
		from awCVTZ or awCVTZ-PP			SCF optimization in XO_4_^–^		
IO_4_^–^	f	1.393	7.984	8.368	1.692	8.215	8.735
	2f	1.393, 4.867	11.360	12.365	1.329, 5.557	11.582	12.564
AtO_4_^–^	f	1.036000	8.384	9.979	1.340	8.975	10.927
	2f	1.036, 2.704	9.975	12.270	0.853, 2.450	10.071	12.404
		from awCVQZ or awCVQZ-PP			SCF optimization in XO_4_^–^		
IO_4_^–^	f	1.470	4.073	4.672	2.779	5.902	6.786
	2f	1.470, 4.102	7.240	8.355	2.200, 8.451	8.162	9.441
AtO_4_^–^	f	1.193	4.294	5.769	1.846	5.396	7.228
	2f	1.193, 2.032	5.499	7.373	1.334, 3.085	5.630	7.559
		from awCV5Z or awCV5Z-PP			SCF optimization in XO_4_^–^		
IO_4_^–^	f	1.500	3.132	3.650	3.264	5.317	6.176
	2f	1.500, 3.439	5.722	6.657	2.560, 9.633	7.261	8.477
AtO_4_^–^	f	1.313	3.224	4.409	2.067	4.043	5.498
	2f	1.313, 2.198	4.084	5.561	1.529, 3.372	4.156	5.665

### 3.2 Correlation Components

It has been known since the late 1990s (e.g., ref (5)) that the lion’s share of the “tight d function effect” in second row elements is recovered at the Hartree–Fock level and that the correlation energy is only weakly affected.

For the XO_4_^–^ series, Table 5 sheds some light on the matter. We present there not only the contributions to the total atomization energy (in kcal/mol) but also the cumulative d- and f-type NAO (natural atomic orbital) populations on the central halogen atom. These latter populations were obtained using the built-in implementation of NBO (natural bond orbital) theory^8^ in MOLPRO 2022.^26^ In Table 6, a breakdown of the correlation contribution into CCSD and (T) can be found.

**Table 5:** Changes in NBO Occupations on the Central Halogen Atom upon the Addition of an Extra d or f Function to the aVTZ(-PP) Basis Set and Increases in the Total Atomization Energy (TAE, kcal/mol) upon Addition of Said Functions

	*Δq*(d)			*Δq*(f)		
	HF-SCF	CCSD(T)_corr_	(T)	HF-SCF	CCSD(T)_corr_	(T)
ClO_4_^–^	0.0405	0.0084	0.0016	0.0004	0.0003	0.0001
BrO_4_^–^	0.0021	0.0005	0.0001	0.0017	0.0004	0.0001
IO_4_^–^	0.0025	0.0000	0.0000	0.0138	0.0021	0.0002
AtO_4_^–^	0.0003	–0.0004	–0.0001	0.0169	0.0047	0.0007

ΔTAE (kcal/mol)						
aVTZ vs aV(T+d)Z, aVTZ-PP vs aVTZ-PP+1f						
ClO_4_^–^	21.205	1.924	0.318	0.717	0.110	0.008
BrO_4_^–^	0.672	0.186	0.015	1.542	–0.120	0.003
IO_4_^–^	0.083	0.023	0.004	7.984	0.377	0.007
AtO_4_^–^	0.071	–0.014	0.011	8.384	1.595	0.181
aVQZ vs aV(Q+d)Z, aVQZ-PP vs aVQZ-PP+1f						
ClO_4_^–^	12.091	1.175	0.204			
IO_4_^–^				4.073	0.600	0.001
AtO_4_^–^				4.294	1.475	0.120
aV5Z vs aV(5+d)Z, aV5Z-PP vs aV5Z-PP+1f						
ClO_4_^–^	2.697	0.184	0.042			
IO_4_^–^				3.650	0.518	0.008
AtO_4_^–^				3.224	1.185	0.112
aV6Z vs aV(6+d)Z						
ClO_4_^–^	0.946	0.039	0.017			
aVDZ vs aV(D+d)Z, aVDZ-PP vs aVDZ-PP+1f						
ClO_4_^–^	40.408	2.380	0.524			
BrO_4_^–^	7.178	0.266	0.110	1.730	–0.008	0.009
IO4_4_^–^				8.882	0.359	0.010
AtO_4_^–^				14.110	3.232	0.254

**Table 6:** Difference between the *E*_corr_CCSD and *E*_corr_(T) Components of the Total Atomization Energy Obtained with the aVTZ(-PP) and aVTZ(-PP)+(d,f) Basis Sets [aVTZ(-PP)-aVTZ(-PP)+(d,f) or awCVTZ-PP in kcal/mol]^a^

	*ΔE*_corr_CCSD	*ΔE*_corr_(T)	*ΔE*_corr_CCSD	*ΔE*_corr_(T)	*ΔE*_corr_CCSD	*ΔE*_corr_(T)	*ΔE*_corr_CCSD	*ΔE*_corr_(T)
	ClO_4_^–^		SO_3_		PF_5_			
aVTZ	REF	REF	REF	REF	REF	REF		
aV(T+d)Z	1.606	0.317	0.359	0.005	0.403	0.057		
aVTZ+1d	1.704	0.321	0.479	0.016	0.486	0.062		
aVTZ+2d	1.915	0.380	0.529	0.023	0.551	0.073		
aVTZ+1f	0.102	0.008	0.220	0.021	0.175	0.005		
aVTZ+2d1f	2.168	0.401	0.861	0.052	0.787	0.082		
awCVTZ	2.013	0.395	0.916	0.071	0.895	0.093		
	BrO_4_^–^		SeO_3_		AsF_5_		KrF_6_	
aVTZ-PP	REF	REF	REF	REF	REF	REF	REF	REF
aVTZ-PP+1d	0.172	0.015	0.135	0.015	0.184	0.014	0.026	0.013
aVTZ-PP+2d	0.183	0.019	0.156	0.018	0.224	0.017	0.015	0.018
aVTZ-PP+1f	–0.123	0.003	–0.071	0.008	0.064	0.005	–0.172	0.029
aVTZ-PP+2d1f	0.034	0.022	0.070	0.026	0.269	0.022	–0.180	0.049
aVTZ-PP+2f	–0.080	0.002	–0.017	0.006	0.152	0.007	–0.174	0.027
aVTZ-PP+2d2f	0.079	0.020	0.129	0.025	0.363	0.025	–0.186	0.047
awCVTZ-PP	0.139	0.057	0.251	0.061	0.544	0.045	–0.589	0.106
	IO_4_^–^		TeO_3_		SbF_5_		XeF_6_	
aVTZ-PP	REF	REF	REF	REF	REF	REF	REF	REF
aVTZ-PP+1d	0.024	0.004	0.068	0.013	0.323	0.018	–0.031	0.005
aVTZ-PP+2d	0.053	0.008	0.107	0.017	0.399	0.023	–0.019	0.009
aVTZ-PP+1f	0.377	0.007	0.010	–0.031	1.261	0.100	0.294	0.070
aVTZ-PP+2d1f	0.413	0.017	0.107	–0.012	1.588	0.122	0.253	0.082
aVTZ-PP+2f	0.945	0.060	0.289	–0.031	2.559	0.193	0.689	0.141
aVTZ-PP+2d2f	0.989	0.071	0.404	–0.010	3.003	0.227	0.633	0.152
awCVTZ-PP	0.757	0.150	0.322	0.035	3.376	0.276	0.006	0.210
	AtO_4_^–^		PoO_3_					
aVTZ-PP	REF	REF	REF	REF				
aVTZ-PP+1d	–0.025	0.011	0.061	0.019				
aVTZ-PP+2d	–0.016	0.013	0.068	0.021				
aVTZ-PP+1f	1.413	0.181	0.882	0.094				
aVTZ-PP+2d1f	1.381	0.199	0.947	0.118				
aVTZ-PP+2f	2.038	0.257	1.406	0.132				
aVTZ-PP+2d2f	2.008	0.276	1.495	0.161				
awCVTZ-PP	1.255	0.372	1.180	0.211				

a Connected doubles, *E*(CCSD) – *E*(SCF) ≡ *E*_corr_CCSD; Noniterative connected triples, *E*(CCSD(T)) – *E*(CCSD) ≡ *E*_corr_(T).

The largest correlation contribution is for the d orbital in ClO_4_^–^, 1.9 kcal/mol, and even that is an order of magnitude less than the HF component. Out of that, 0.3 kcal/mol is accounted for by the triples contribution. These correlation contributions are nearly cut in half for the next basis set in the series, aug-cc-pVQZ vs aug-cc-pV(Q+d)Z, and again the SCF contribution exceeds that of correlation by an order of magnitude.

While in computational thermochemistry protocols like W4 theory,^40^ which covers first- and second-row molecules, we have always striven to include tight d functions also for the CCSD(T) correlation steps, the post-CCSD(T) steps always omit them (in part because of the steep computational cost scaling of CCSDT, CCSDT(Q), and CCSDTQ). A very recent study by Karton^41^ reconsiders this aspect and indicates that the inclusion of tight d functions even in these steps may have enough of an effect to be significant in high-accuracy computational thermochemistry (see Karton^42^ for a very recent review).

For periodate and perastatate, the tight f contribution is in the 8 kcal/mol range, albeit especially for perastatate with a more noticeable correlation contribution. Still, the HF contribution dominates by far.

As a rule of thumb, going up from aVTZ to aVQZ appears to cut the tight d or tight f contribution in half. Going one step further up the hierarchy to aV(5+d)Z and aV(6+d)Z, the tight d contribution for ClO_4_^–^ definitely tapers off smoothly, but contributions in excess of 3–4 kcal/mol are still seen with aV5Z-PP basis sets for IO_4_^–^ and AtO_4_^–^.

Comparison of the NBO charges with and without the extra functions reveals that, at the HF level, the aV(T+d)Z basis set for ClO_4_^–^ has about 0.04 electrons more d occupation than without the extra d, while the extra tight f functions on iodine and astatine cause increases of 0.014 and 0.017 for IO_4_^–^ and AtO_4_^–^, respectively. The contribution of correlation is a factor of 3–7 smaller, and out of that, (T) accounts for a negligible fraction. The latter means that CCSD will be generally enough for differential NBO analysis of this type—which is quite convenient, as CCSD densities are both much more economical than CCSD(T) and available in more electronic structure programs (notably, Gaussian).

For DFT calculations on heavier elements, the popular Weigend–Ahlrichs “def2” basis sets^17^ are widely used owing to their availability for all elements through radon. Weigend and Ahlrichs^17^ mention in passing that two additional tight f functions are needed for heavier p-block elements due to what they deem to be “core polarization”. Now if this interpretation were correct, then the need for the tight f functions would go away if the subvalence electrons were all replaced by a large-core ECP. We thus repeated the IO_4_^–^ calculations using Martin and Sundermann’s large-core SDB-aug-cc-pVTZ and QZ basis sets,^43^ which for practical applications were superseded by the small-core “official” (aug-)cc-pVnZ-PP basis sets.^30,44^ Despite iodine now having all 46 core electrons, [Kr](4d)^10^, “rolled into” the ECP, it turns out we need the tight f functions just as much, which rules out core polarization. Repeating a numerical experiment that was “left on the cutting room floor” of the final published version of the 1998 SO_2_ paper,^5^ we replaced the sulfur core electrons in SO_2_ and SO_3_ with an ECP10MWB pseudopotential (while decontracting the s and p functions of the sulfur aug-cc-pVnZ basis set to avoid contraction mismatch with the ECP) and found that the need for tight d functions was likewise essentially the same as for the all-electron calculation. (See also the very recent large-core ccECP-cc-pV(n+d)Z basis sets of Hill and co-workers.^45^)

### 3.3 Density Functional Theory

It stands to reason that the same observations made above for Hartree–Fock would also apply to other independent-particle models, specifically to DFT. For the tight d functions in the second row, this was indeed first spotted^4^ at the B3LYP and CCSD(T) levels and later confirmed^5^ at the HF level.

For the sake of completeness, we have repeated our analysis for the PW6B95 hybrid meta-GGA functional. As can be seen in the Supporting Information, our observations at the PW6B95 level are fundamentally the same as at the HF level.

## 4 Conclusions

We have examined the effect of tight d and f functions in aug-cc-pVnZ (or aug-cc-pV(n+d)Z) basis sets on SCF and post HF contributions to the total atomization energies and vibrational frequencies for p-block fluorides and oxides. From the present study, we can conclude that the need for added high-exponent d functions to the second-row p-block elements (as done in the (aug-)cc-pV(n+d)Z basis sets^16^) has a direct (albeit milder) parallel in the fourth and fifth row, but now in terms of high-exponent f functions.

The effect is linked to the 3d, 4f, and 5f virtual orbitals of second, third, and fourth row elements approaching the valence shell as one approaches, respectively, the first-row transition metals, the lanthanides, and the actinides. Additionally, with increasing oxidation states, these orbitals will sink still lower and become still better back-donation acceptors from halogen and chalcogen lone pairs.

In the third row p-block, the 4f is still too remote, while the 4d is adequately covered by the basis functions needed to describe the 3d subvalence orbital.

An alternative explanation in terms of core polarization was refuted by means of large-core ECP calculations in which there are no core orbitals left to polarize.

## Supporting Information Available

Spreadsheet with XYZ coordinates and all calculated ΔTAE values and harmonic frequencies at the PW6B95 levels of theory, as well as the full ref (25) (XLSX)
